# Robotic-assisted laparoscopic cholecystectomy with indocyanine green fluorescent cholangiography and intraoperative cholangiogram for patients with acute and chronic cholecystitis: a case series

**DOI:** 10.1093/jscr/rjaf592

**Published:** 2025-09-09

**Authors:** Saamia Shaikh, Nawras Radwan, Tamara Cheski, Franz Yanagawa

**Affiliations:** Department of Surgery, St. Joseph’s University Medical Center, 703 Main Street, Paterson, NJ 07503, United States; Department of Surgery, St. Joseph’s University Medical Center, 703 Main Street, Paterson, NJ 07503, United States; Department of Surgery, St. Joseph’s University Medical Center, 703 Main Street, Paterson, NJ 07503, United States; Department of Surgery, St. Joseph’s University Medical Center, 703 Main Street, Paterson, NJ 07503, United States

**Keywords:** robotic cholecystectomy, intraoperative cholangiogram, IOC, ICG, cholecystitis

## Abstract

Indocyanine green fluorescent cholangiography (ICG) and intraoperative cholangiography (IOC) are both useful during cholecystectomy. Laparoscopic cholecystectomy with IOC is commonly performed in various situations; however, there have been concerns with performing IOC during robotic cholecystectomy such as operating room set up and increased operative time due to docking and undocking of the robot. We argue if IOC is readily available and is possible, safe, and not extremely time consuming, that it should be utilized in conjunction with indocyanine green fluorescent cholangiography, instead of preoperative magnetic resonance cholangiopancreatography (MRCP). This could potentially decrease overall cost and the hospital length of stay. We report our experience with ten cases and describe our operative technique.

## Introduction

Indocyanine green fluorescent cholangiography (ICG) and intraoperative cholangiography (IOC) have both been used during cholecystectomy. Traditionally, IOC has been used during difficult cholecystectomies to help delineate extrahepatic biliary anatomy [1]. It is also useful to assess for common bile duct (CBD) stones. ICG has similarly been used during difficult dissections during cholecystectomy. It is a newer modality which aids in delineating the biliary anatomy during cholecystectomy but is not the equivalent of the classic cholangiography. Unlike IOC, ICG is radiation-free and is usually administered intravenously. It also does not require extra personnel (i.e. X-ray technician to maneuver the C-arm).

With the advent of robotic-assisted cholecystectomy, it seems that the use of magnetic resonance cholangiopancreatography (MRCP), and, if positive for choledocholithiasis, endoscopic retrograde cholangiopancreatography (ERCP) followed by simple cholecystectomy has become mainstream. While some argue that MRCP should be performed routinely before cholecystectomy to detect CBD stones, identify biliary anatomy, and for preoperative planning [2], we argue that if IOC is readily available and is possible, safe, and not extremely time consuming, that it should be utilized in conjunction with ICG, instead of preoperative MRCP. MRCP can be costly, increase hospital length of stay, and may delay definitive treatment for cholecystitis [3, 4]. Although IOC can increase operative duration, it can be performed safely and may eliminate the need for ERCP and sphincterotomy if CBD exploration is performed during the index procedure. Given the positive association between biliary sphincterotomies and biliary cancers [5], it may be worthwhile to perform IOC and CBD exploration, when indicated, preventing the need for ERCP and sphincterotomy.

## Case series

**Figure 1:**
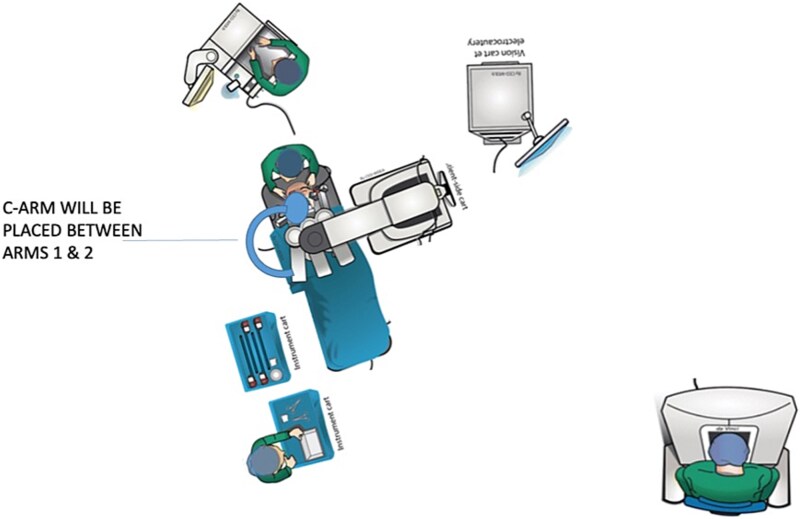
Diagram of set up in operating room.

Anecdotally, we have found that the key step is to have all the equipment present in the operating room at the beginning of the case. After the patient is prepped and draped, the table is moved toward the back wall of the OR, away from the anesthesia set up, and turned diagonally. This allows the C-arm to come in from the patient’s right while the robot is docked from the left side (Fig. 1).

We use the 4-arm approach to the cholecystectomy. After the ports are placed, the robot is docked and targeted and then arms 3 and 4 are moved to the most lateral position, corresponding to the “X” on the FLEX positioning of the arm. The surgery is then continued as per usual. Once the critical view of safety (CVS) is nearly complete, the X-ray technician is called in preparation for positioning. When the CVS is completed and time out to identify the proper structures is complete, arms 1 and 2 are moved out to the extreme position of “F” on the FLEX positioning of the arms (or as close as possible) and folded to accommodate the C-arm. The hook cautery is removed from the 4th arm and subsequently replaced with a grasper (e.g. Force Bipolar, Cadiere, etc.). The first and second ports are then used for a standard laparoscopic grasper for the apex of the gallbladder and Kumar Clamp, respectively. The catheter is then directed into the cystic duct with the help of the grasper in the 4th arm. Cholangiogram is then shot in the standard fashion without the need for undocking the robot.

### Case 1

**Figure 2:**
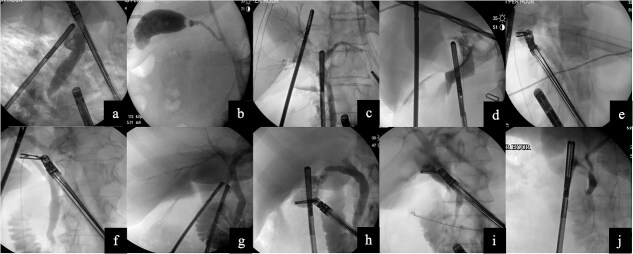
Images from intraoperative cholangiograms—(a) Case 1, (b) Case 2, (c) Case 3, (d) Case 4, (e) Case 5, (f) Case 6, (g) Case 7, (h) Case 8, (i) Case 9, and (j) Case 10.

A 48-year-old female with cholelithiasis presented to the emergency department (ED) complaining of a 1-day history of mild, constant abdominal pain associated with nausea and vomiting. On physical exam, she had moderate right upper quadrant and epigastric tenderness. Ultrasound revealed cholelithiasis with an impacted calculus in the neck of the gallbladder and a dilated CBD of 10 mm. The decision to forgo MRCP was made. Instead, the patient underwent a robotic laparoscopic cholecystectomy with ICG and IOC. The cholangiogram did not show any filling defects (Fig. 2a). She was seen in the office ~2 weeks later and made an uneventful recovery.

### Case 2

A 55-year-old obese male presented to the ED complaining of sudden-onset midsternal and epigastric abdominal pain that radiated to his back. Labs revealed a leukocytosis of 16.5, lipase of 27 679, aspartate aminotransferase (AST) of 124, and alanine aminotransferase (ALT) of 114, alkaline phosphatase (alk phos) of 93, and a total bilirubin level of 1.3. The patient was initially not optimized for a surgical procedure and had a cholecystostomy tube placed. Subsequently, he underwent a robotic cholecystectomy with ICG, IOC, removal of the cholecystostomy tube, and Blake drain placement. The cholangiogram was negative for any filling defect (Fig. 2b). The patient tolerated the procedure without any issues and was discharged home. He made an uneventful recovery.

### Case 3

The patient was an 84-year-old female who presented to the ED with sharp, epigastric abdominal pain. Labs revealed a leukocytosis of 12.5, lipase of 13 365, AST of 702 and ALT of 491, alk phos of 56, and a total bilirubin level of 2.1. Ultrasound revealed cholelithiasis. The patient underwent a robotic cholecystectomy with ICG and IOC. The cholangiogram did not show any filling defects (Fig. 2c). The patient tolerated the procedure without any issues and was discharged home.

### Case 4

A 24-year-old female with morbid obesity presented to the ED with right upper quadrant abdominal pain. Labs were significant for leukocytosis of 12.3. Ultrasound revealed cholelithiasis and a dilated CBD of 8 mm. The patient underwent a robotic cholecystectomy with ICG and IOC. The cholangiogram showed a sharp cut-off shortly after the cystic duct, suspicious for a stone at the confluence of the cystic duct and CBD (Fig. 2d). Postoperative ERCP revealed sludge in the CBD. Complete removal was accomplished by biliary sphincterotomy and balloon extraction. Post-sphincterotomy cholangiogram was normal. The patient was discharged and seen in the clinic 2 weeks later and made an uneventful recovery.

### Case 5

The patient was a 51-year-old female who presented to the ED with persistent postprandial right upper quadrant abdominal pain. Labs were remarkable for AST of 293 and ALT of 206. Ultrasound was equivocal for acute cholecystitis and revealed an intraluminal filling defect. She underwent a robotic cholecystectomy with ICG and IOC. The cholangiogram showed proper retrograde filling of the left and right hepatic ducts and adequate anterograde visualization of the CBD (Fig. 2e). The patient was discharged and made an uneventful recovery without the need for an ERCP.

### Case 6

The patient was a 61-year-old obese female who presented to the ED with a 1-day history of constant right upper quadrant pain associated with nausea and vomiting. Labs revealed a leukocytosis of 16.9, lipase of 11 211, AST of 125, ALT of 123, alk phos of 131, and total bilirubin level 0.7. Ultrasound revealed cholelithiasis and sludge. Computer tomography of the abdomen and pelvis revealed mild diffuse peripancreatic edema, suspicious for acute pancreatitis. Patient underwent robotic cholecystectomy with ICG and IOC. Cholangiogram revealed CBD dilation but no stones (Fig. 2f). She was seen two weeks later in the office and made an uneventful recovery.

### Case 7

The patient was a 35-year-old male with cholelithiasis who presented with a 5-day history of right upper quadrant abdominal pain. Labs were remarkable for a mild elevation of the ALT at 73. Ultrasound revealed cholelithiasis and a CBD of 5.8 mm. Patient was taken for robotic-assisted laparoscopic cholecystectomy with ICG and IOC. IOC was negative for any filling defects or strictures (Fig. 2g). Patient was discharged and recovered well with no complications.

### Case 8

The patient was a 22-year-old female with cholelithiasis who presented with 2 months of intermittent nausea and epigastric pain. Previous ultrasound revealed a contracted gallbladder with cholelithiasis and a CBD of 6.8 mm. Labs were remarkable for AST of 210 and ALT of 188. Patient was taken for robotic cholecystectomy with ICG and IOC. IOC was negative for any filling defects or strictures and showed contrast draining into the duodenum (Fig. 2h). Patient was discharged and was seen a week later for postoperative visit; she recovered well with no complications.

### Case 9

The patient was a 38-year-old female who presented to the ED with a 1-day history of abdominal pain with associated nausea and vomiting. Ultrasound was suspicious for acute cholecystitis and the CBD was 5.86 mm. Patient underwent robotic-assisted laparoscopic cholecystectomy with ICG and IOC. IOC was negative for filling defects, strictures, and showed contrast in the duodenum as well as the hepatic ducts (Fig. 2i). Patient was discharged postoperatively and was seen 2 weeks later for a postoperative visit; she recovered well with no complications.

### Case 10

The patient was a 67-year-old female with a recent hemorrhagic stroke who presented with a 1-week history of abdominal pain and was found to have gallstone pancreatitis. The medicine team ordered an MRCP which revealed cholelithiasis with gallbladder wall thickening without any evidence of biliary dilatation or CBD stones. She underwent robotic cholecystectomy with ICG and IOC. IOC was negative for filling defects (Fig. 2j). Patient recovered uneventfully.

## Discussion

Both IOC and ICG can be used during laparoscopic or robotic-assisted cholecystectomy. They share a similar purpose in identifying anatomy and aiding the surgeon in visualizing key structures.

ICG is a dye that fluoresces when exposed to near-infrared light and is viewed around a maximum peak of 832 nm [6]. This enables surgeons to assess critical structures in real time and provides an anatomical distinction that is otherwise unobtainable to the naked eye. ICG is also water-soluble, and when injected intravenously, it binds to proteins that undergo hepatic clearance [7]. The ICG is eventually excreted in the bile, which is useful for outlining the biliary tree. This provides great detail of the hepatic anatomy and has become incredibly valuable in hepatobiliary surgery, including cholecystectomy.

IOC involves radiographic imaging that utilizes contrast and fluoroscopic pictures in real time to visualize the gallbladder, cystic duct, CBD, hepatic ducts, forward flow of contrast into the duodenum, and any potential abnormalities such as filling defects. For example, IOC highlights the presence of stones or strictures in the biliary system and plays a role in assisting with surgical decision-making, such as the decision to perform a CBD exploration for CBD stones [8].

ICG, like IOC, can allow for visualization of the cystic duct, CBD, and common hepatic duct [9]. Compared to white light alone, some have found that ICG was able to identify critical anatomical structures quicker [10]. Likewise, some authors have found that operative duration of cholecystectomy with ICG was significantly shorter than cholecystectomy with IOC [9]. In addition to ICG’s ability to delineate critical structures faster, ICG does not require making an extra incision, is cost-friendlier, and is radiation-free [10]. Moreover, ICG can be used safely in pregnant patients [10]. Furthermore, in order to perform an IOC, it is necessary to incise what is believed to be the cystic duct, and this itself could be a source of injury to the CBD or common hepatic duct [9]. Some have suggested that ICG could potentially replace IOC for biliary mapping [9]. However, unlike IOC, ICG has not effectively detected CBD stones [11]. As such, while useful in delineating the anatomy during operative dissection, cannot replace IOC when evaluating for choledocholithiasis or biliary stricture, for example.

As with our cases, some authors have demonstrated that the use of IOC does not necessarily require the undocking of the robotic arms. Acho *et al.* shared their technique whereby they were able to position the robotic arms in a manner that allowed the C-arm to be placed above the robotic ports [12]. This included inserting a 5-mm port in the right midclavicular line below the costal margin for the cholangiogram catheter and swinging arms 1 and 2 to the patient’s right and arms 3 and 4 to the patient’s left. They also described dropping all the robotic elbows toward the patient’s abdomen using the patient clearance button as well as raising the tips of the instruments toward the abdominal wall to lower the instrument insertion access point. After this, the C-arm can be introduced between arms 2 and 3 without any undocking required. This technique is efficient and does not add much additional time to the operative duration.

IOC in addition to ICG can be used instead of preoperative MRCP to safely identify biliary anatomy and any CBD abnormalities (e.g. filling defects, strictures). We argue that performing IOC does not prolong intraoperative times significantly and it is feasible and safe. As with laparoscopic cholecystectomies, we believe that performing robotic cholecystectomies with intraoperative cholangiogram ± CBD exploration should be performed instead of doing a preoperative MRCP and ERCP (when indicated). MRCP can be costly, increase hospital length of stay, and can delay definitive management of cholecystitis and as such should only be reserved for select patients.
